# #BlackBreastsMatter: Process Evaluation of Recruitment and Engagement of Pregnant African American Women for a Social Media Intervention Study to Increase Breastfeeding

**DOI:** 10.2196/16239

**Published:** 2020-08-10

**Authors:** Cassy Dauphin, Nikia Clark, Renee Cadzow, Frances Saad-Harfouche, Elisa Rodriguez, Kathryn Glaser, Marc Kiviniemi, Maria Keller, Deborah Erwin

**Affiliations:** 1 Cancer Prevention and Control Roswell Park Comprehensive Cancer Center Buffalo, NY United States; 2 Department of Health Services Administration D'Youville College Buffalo, NY United States; 3 Department of Health, Behavior and Society University of Kentucky Lexington, KY United States; 4 Department of Community Health and Health Behavior State University of New York at Buffalo Buffalo, NY United States

**Keywords:** breastfeeding, breast cancer education, African American mothers, Facebook, mobile phone, social media

## Abstract

**Background:**

In the United States, there are lower rates of breastfeeding among African American mothers, particularly those who are younger women. Recent epidemiological studies have shown a strong association of more aggressive types of breast cancer (estrogen receptor negative) among African American women, with a higher risk in African American women who did not breastfeed their children.

**Objective:**

This study aims to describe the process evaluation of recruitment and educational strategies to engage pregnant African American participants for a pilot study designed to determine whether social media messaging about breast cancer risk reduction through breastfeeding may positively influence breastfeeding rates.

**Methods:**

This pilot study is conducted in collaboration with a local Women, Infants, and Children (WIC) organization and hospital and prenatal clinics of a local health care network. To engage African American women to enroll in the study, several methods and monitoring processes were explored, including WIC electronic text-based messages sent out to all phones of current WIC recipients (referred to as *e-blasts*); keyword responses to texts from flyers and posters in local community-based organizations, hospitals, and prenatal clinics; keyword responses using electronic links posted in established Facebook groups; and *snowball* recruitment of other pregnant women by current participants through Facebook. Once enrolled, participants were randomized to 2 study conditions: (1) an intervention group receiving messages about breast cancer risk reduction and breastfeeding or (2) a control group receiving breastfeeding-only messages. Data were obtained through electronic monitoring, SurveyMonkey, qualitative responses on Facebook, focus groups, and interviews.

**Results:**

More than 3000 text messages were sent and received through WIC e-blasts and keyword responses from flyers. A total of 472 women were recruited through WIC e-blast, and 161 responded to flyers and contacts through the local health care network, community-based organizations, Facebook, and friend referrals. A total of 633 women were assessed for eligibility to participate in the study. A total of 288 pregnant African American women were enrolled, consented, and completed presurvey assessments (102.8% of the goal), and 22 participants attended focus groups or interviews reporting on their experiences with Facebook and the educational messages.

**Conclusions:**

This process evaluation suggests that using electronic, smartphone apps with social media holds promise for both recruitment and conduct of health education intervention studies for pregnant African American women. Providing messaging and resources through social media to reinforce and educate women about breastfeeding and potentially provide lactation support is intriguing. Convenience (for researchers and participants) is an attribute of social media for this demographic of women and worthy of further research as an educational tool.

**Trial Registration:**

ClinicalTrials.gov NCT03680235; https://clinicaltrials.gov/ct2/show/NCT03680235

## Introduction

### Background

Over the past 25 years, African American (AA) mothers have had consistently lower breastfeeding rates than any other group of women in the United States [1]. Only 74.0% of AA infants are ever breastfed compared with 86.6% of non-Hispanic White infants and 82.9% of Hispanic infants [2]. Epidemiological evidence shows that this disparity may negatively impact health and increase breast cancer risk in AA women [3]. Given the combination of evidence for racial disparities in lactation patterns and reduced breast cancer risk with breastfeeding for mothers, AA women may be disproportionately impacted by multiple negative health problems—with a strong opportunity for intervention. Although developing an educational intervention to impact this behavior change is challenging, a recent systematic review of interventions to enhance breastfeeding rates suggests that multilevel interventions can be successful. There is a significant need to explore social media communication, text messaging, and the internet to provide tools and methods for reaching and engaging AA mothers [1]. Given the evidence of health benefits to mothers and infants from breastfeeding, exploring novel interventions for reducing these socially patterned disparities is a significant scientific goal and the focus of this paper.

### Breastfeeding and Breast Cancer Risks

**“**Breastfeeding rates in the United States (US) are considered socially patterned. Previous research has documented startling racial and socioeconomic disparities in infant feeding practices [4].” Nationally, AA infants and mothers are approximately 12% less likely to have the benefit of breastfeeding compared with White infants and mothers [2,5,6]. The most recent data as of 2017 from the New York State Department of Health indicate that breastfeeding initiation (*ever breastfed*) has increased to 84% in the AA population compared with 80.1% of White counterparts, but this does not include exclusive breastfeeding [7]. Moreover, by 6 months, the breastfeeding rates for babies were only 38% for AA and 44.5% for whites [7].

Recent epidemiological research suggests that choosing formula feeding over breastfeeding may have a significant impact on the risk of developing aggressive, triple-negative breast cancer (TNBC) in AA women [3]. Research findings reported by Palmer et al [3] showed that parity—formerly considered a risk reduction factor—actually *increases the risk* of TNBC in AA women. However, these breast cancer risks can be ameliorated in AA women if they initiate breastfeeding, and further research on the role of duration continues to be studied [3]. These results suggest that promoting breastfeeding in AA women may be an effective tool for reducing the occurrence of TNBC, which disproportionately contributes to breast cancer mortality for these women [3]. Therefore, encouraging breastfeeding among AA mothers offers a strong opportunity for intervention, given the combination of evidence for reduced cancer risk with breastfeeding and the overall national racial disparities in lactation patterns.

### Barriers to Breastfeeding

Multiple barriers to breastfeeding among AA mothers have been reported in literature [1]. Primary issues include the burden of breastfeeding by women who work in jobs with little flexibility, unsupportive employers, and having limited maternity leave [4,8]. In addition, the social context for breastfeeding is weakened because AA women may not discuss the benefits of breastfeeding because of a lack of understanding and awareness [9,10]. Some women may have cultural discomfort regarding negative historical images as a result of the legacy of slavery [1,11,12]. Survey data collected from 225 middle and high school youth aged 13 to 18 years in Erie County, New York, showed that 62% of youth agreed or strongly agreed that “Breast milk is the best food for baby,” but only 28% agreed or strongly agreed that “Breasts are meant for breastfeeding,” and 36% reported this behavior as annoying. Young people exposed to formula feeding still outnumber those exposed to breastfeeding. These data demonstrate the challenges and limitations of changing cultural contexts to support breastfeeding. Personal experience and public or media exposure are statistically correlated with future intent to breastfeed [13]. Therefore, the educational intervention messages for this study included images aimed at positively impacting feelings and intent to breastfeed, and qualitative assessments included family members and partners of the mothers to explore social context.

### Social Media and Smartphone Use for Reaching AA Women

To reach pregnant AA women, investigators chose to explore social media platforms for both study recruitment and intervention engagement, as smartphone and social media use are prevalent in this demographic. Research data show that the rate of social media use by all Americans is currently 69%, which is the same use rate among AAs [14]. The most frequently used social media platforms are Facebook and Instagram. The usage of these two social media platforms varies by age. Research indicates that 81% of young adult women aged between 18 and 29 years use Facebook, and 64% of this same demographic use Instagram [15]. For many users, social media has become part of their daily routine, especially as most people have access to smartphones. The frequency of use by young adults aged between 18 and 29 years who check their Facebook or Instagram account daily is 72% [15]. Nearly three-fourth of AAs use smartphones to access social media compared with approximately 66% using desktop or laptop computers [14]. Facebook reported that the majority of users use the platform to post personal ideas, opinions, life events, and milestones (ie, graduations, vacations, employment, weddings, pictures of family, friends, or selfies) [15]. Moreover, social media communication methods may help address the reported lack of personal support [16] and low self-efficacy related to breastfeeding [1].

It is notable that income levels no longer present a major challenge in owning a smartphone, as 63% of low-income (<US $30,000 a year) AAs are owners of smartphones. However, low-income smartphone users who are AA are about twice as likely as whites to have their smartphone use canceled, or service interrupted because of the expense [14]. These interruptions with cell phones may create challenges when delivering services to low-income groups, although web-based social media platforms such as Facebook provide a unique mechanism of communication that continues despite cell service interruption.

Findings from prior research demonstrated similar rationales for the use of the technology in study recruitment for first-time AA mothers and for the use of social media to disseminate health education information on breastfeeding [17]. Social media access and use as an effective social support mechanism in pregnancy and postpartum have been tested by others [18]. Baker and Yang [18] found that social media present the opportunity to educate new mothers from the comfort of their home 24 hours a day.

This paper offers a process evaluation focused on the recruitment and study implementation techniques for a pilot breastfeeding intervention via smartphones and social media platforms among AA mothers participating in Western New York. As part of this larger pilot study, it was important to explore the acceptance and process of using social media and electronic apps to engage pregnant AA women with specific health messaging. This study focused on describing the methodological processes, benefits, and limitations for recruiting and engaging women through a social media conduit regarding breastfeeding and breast cancer risk reduction.

## Methods

### Intervention

**Figure 1:**
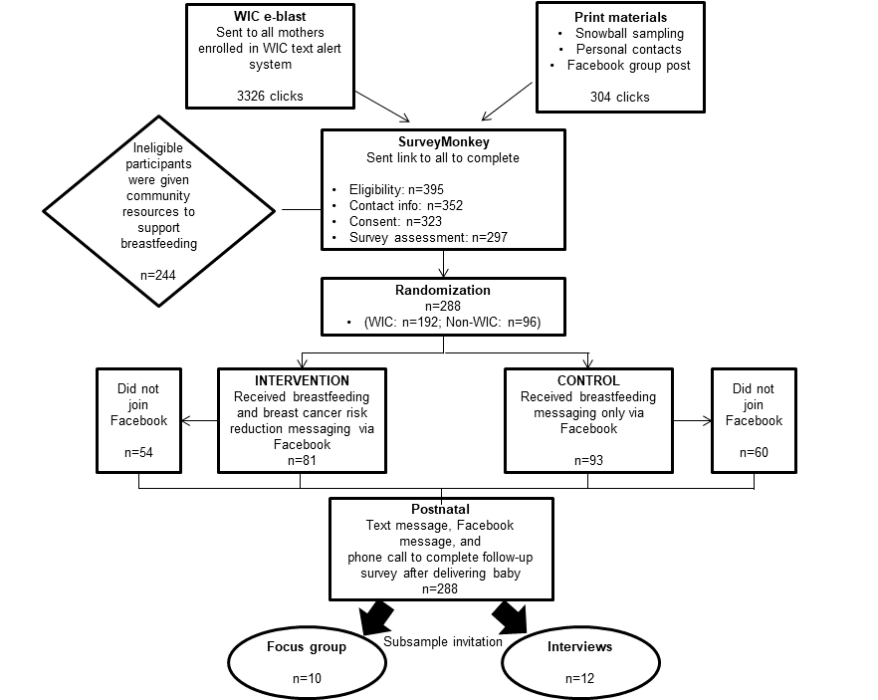
Study schema. WIC: Women, Infants, and Children.

The pilot study intervention was centered on enrolling eligible participants (AA, pregnant, and English speaking) into a private Facebook platform, and participants were randomized into either a control arm in which they received breastfeeding-only messaging or an intervention arm that received breastfeeding and breast cancer risk reduction messaging (Figure 1). Participants were asked to complete pre- and postbirth assessments and a smaller subgroup of mothers and their family members were recruited to participate in either focus groups or semistructured interviews postpartum. The goal was to obtain opinions from the mothers on the content presented in the Facebook group as well as the feasibility and effectiveness of using smartphone technology and Facebook for education and promotion of breastfeeding, with a specific aim of determining any outcome differences by specific message content (breastfeeding vs breast cancer risk reduction and breastfeeding).

Family members and partners of the mothers were included in the qualitative assessments to obtain pertinent social influences in the context of mothers’ experiences. Focus groups and interviews included mothers and their support persons (family, friends, or partners) to discuss the content presented in the Facebook group. This study was registered in ClinicalTrials.gov, under registration number NCT03680235.

### Study Participants

The study population consisted of pregnant AA mothers residing in Erie and Niagara County, especially those served by Women, Infants, and Children (WIC). As the WIC program in Erie County serves a significant proportion of the pregnant AA women in the area, we partnered with WIC to inform their clients about the study. When the study was designed (2016), the Erie/Niagara WIC assisted women with approximately 2000 births per year; however, data are not available by race [19].

### Participant Demographics

Of the study participants, approximately 25.3% (73/288) completed high school or received a general educational development (GED) certificate, 33.3% (96/288) completed some college, and 12.2% (35/288) were college graduates. In addition, 55.2% (159/288) of the mothers reported that they were employed, approximately 56.6% (163/288) reported making less than US $20,000 per year, 13.5% (39/288) earned US $20,000 to US $29,999, and 13.5% (39/288) earned US $30,000 to US $49,999, with 1.0% (3/288) earning more than US $50,000, and 15.3% (44/288) declined to answer. Moreover, 70.8% (204/288) of the mothers were never married; 24.0% (69/288) were married or partnered; and 4.9% (14/288) were divorced, widowed, or separated. A total of 22.6% (65/288) of women reported being breastfed as a child. Overall, 26.7% (77/288) of women reported this as their first pregnancy, 20.8% (60/288) reported this as their second pregnancy, 21.2% (61/288) as their third pregnancy, and 31.3% (90/288) as their fourth or more pregnancy. Approximately, 53.8% (155/288) of the women reported the age of their first pregnancy being 19 years or younger, 36.5% (105/288) reported first pregnancy between the ages of 20 and 25 years, 5.6% (16/288) reported first pregnancy between the ages of 26 and 29 years, and 2.1% (6/288) reported first pregnancy at 30 years and older (Table 1).

**Table 1:** Demographics.

Demographic categories		Values, n (%)
**Age (years)**		
	14-17	3 (1.0)
	18-24	101 (35.1)
	25-34	149 (51.4)
	35-44	35 (12.2)
**Currently employed**		
	Yes	159 (55.2)
	No	127 (44.1)
	Not reported	2 (0.7)
**Marital status**		
	Single or never married	204 (70.8)
	Widowed, divorced, or separated	14 (4.9)
	Married	69 (24.0)
	Not reported	1 (0.3)
**Education**		
	Less than high school	29 (10.1)
	High school or general educational development certificate	73 (25.3)
	Some college	96 (33.3)
	College degree	35 (12.2)
	Not reported	55 (19.1)
**Income (US $)**		
	<20,000	163 (56.6)
	20,000-29,999	39 (13.5)
	30,000-39,999	26 (9.0)
	40,000-49,999	13 (4.5)
	>50,000	3 (1.0)
	Not reported	44 (15.2)
**Age of first pregnancy (years)**		
	<19	155 (53.8)
	20-25	105 (36.5)
	26-29	16 (5.6)
	>30	6 (2.1)
	Not reported	6 (2.1)
**Breastfed as a child**		
	Yes	65 (22.6)
	No	153 (53.1)
	Not reported	70(24.3)

### Recruitment and Assessment Procedures

To engage AA women to enroll in the study, the following methods and monitoring processes were explored: (1) WIC text-based *e-blast* messages were sent to all WIC enrollees at specified clinic sites, (2) in-person recruitment was conducted at prenatal clinics and community-based organization events using keyword response giving participants web links to eligibility criteria and consent documents, (3) keyword response text messages were monitored through Telerivet (Telerivet, Inc) based on flyers and posters in the hospital and WIC office, (4) Bitly (Bitly.com) was used to track *clicks* on the link navigating women to the web-based SurveyMonkey (SVMK Inc) surveys, (5) brochures and posters in prenatal clinics were distributed with electronic links described, (6) recruitment of pregnant women from established support groups through Facebook, and (7) recruitment of other pregnant women by current participants through Facebook was encouraged. Once women consented and completed the prenatal survey, they were randomized to the 2 study conditions and 2 private Facebook groups. Data were obtained through electronic monitoring, SurveyMonkey, qualitative responses on Facebook, focus groups, and interviews.

These recruitment processes used 4 computer-based mobile messaging platforms. The first mobile messaging platform was operated by WIC and was used to disseminate the initial enrollment text through bulk messaging referred to as an *e-blast*. The second app was used to receive enrollment text messages referred to as *keyword responses* and was monitored through Telerivet by study staff. Telerivet is a web-based mobile text messaging app that serves as a messaging command center and allows program owners to send *text blasts* or bulk messaging to multiple participants who would receive the text individually (ie, not group texts). Through this app, study staff were able to streamline communication by using features such as message scheduling or staggering, which allows the scheduling of messages for times or recurring intervals. This app allowed staff to schedule polls via text messages in which participants could respond either by numeric or alphabetic responses, eliminating the need for manual data entry. This app also had an automatic stop function that allowed participants to *opt out* and stop receiving messages.

Bitly is a link management platform that was used to track the amount of interest in the study through *clicks* on the link that navigated women to the web-based survey. Participants could click the e-blast–delivered link multiple times to be able to access and return to the survey. SurveyMonkey was the fourth web-based software used to collect survey responses from the women through their mobile devices and build the study database.

The initial recruitment request was sent out through an *e-blast* to all WIC participants who served and registered at urban Buffalo WIC clinic sites that included the highest proportion of AA clients (Figure 1). The monthly e-blast was sent through the WIC-owned and operated mobile messaging platform to all mothers aged ≥18 years and enrolled at the specified sites who were currently pregnant and due to deliver within 3 months, with the intent to enroll women and allow them to receive Facebook messaging for at least two to three months before delivery. The clients’ mobile phone numbers were an existing part of the WIC client database and used by WIC for various communication processes, so the study team did not have to have clients’ permission to contact individuals. The e-blast message briefly introduced the study to the clients and gave them a link to explore it in more depth (including eligibility criteria and consent). As WIC does not report data by race, e-blast messages were sent to all clients from the selected clinics regardless of race, and eligibility was assessed after they responded. Recruitment text messages were delivered monthly to cohorts of women based on their expected delivery month over 3 months (ie, May-July). Each woman received up to 3 recruitment text messages from the WIC’s mobile messaging platform.

On receiving the text on their smartphones, WIC participants could opt to click on a link to connect them to the study eligibility criteria questions (eg, pregnancy status: due date, WIC enrollment status, race: AA, primary language: English). WIC clients who did not meet the study eligibility criteria received the following message: “Thank you but you are not eligible for this study” and were directed to other links with information about healthy pregnancy practices and community resources such as the Durham Baby Café, a local Center for Teens, Moms & Kids, and WIC Breastfeeding Partners. Eligible participants were directed to the electronic consent form, which required one click at the bottom of the form for *Agree*. The study was approved by the Roswell Park Cancer Institute institutional review board (IRB) as minimal risk.

To be more inclusive of pregnant women aged <18 years (New York State WIC did not allow recruitment of WIC clients aged <18 years for research), the study team partnered with additional obstetrics and gynecology (OB/GYN) offices in the urban area and community-based organizations focused on delivering services to pregnant women. Posters and flyers to promote the study were displayed and disseminated at all participating recruitment sites as well as in-person recruitment by study staff visiting the clinics. The poster and flyers contained a keyword response, “OurBreastsMatters,” which participants were requested to text to a long-code 10-digit local phone number. For consistency, posters, flyers and original e-blasts from WIC used a race-neutral study name because WIC was not able to send messages to only AA clients. After meeting eligibility criteria, participants were directed to the BlackBreastsMatter site for further surveys and Facebook intervention. Similar to the WIC enrollees, participants received an automated response that contained the link to opt in to the study and connect them to the eligibility criteria. These methods allowed participants to initiate the process for enrolling in the study while having the ability to complete the survey at their convenience. In addition, a final strategy consisting of a snowball sampling approach was also used to recruit additional participants by offering this study’s participants an incentive to recruit other pregnant friends and relatives using the keyword response approach.

All study participants received both weekly and monthly scheduled text messages regarding their status in the project as well as their current use of the resources allocated by our partners. Telerivet allowed study staff to streamline participant contact and vary the amount of contact for participants at different intervals in the study.

### Data Collection Procedures

Using SurveyMonkey, participants were asked to complete eligibility criteria, social media use, demographics, study consent, and pre- and postpartum surveys from smartphones. Once the participants consented, they were asked to complete a 73-question preintervention baseline survey on their smartphone. Participants could return to the survey if they did not choose to complete it immediately. Participants were incentivized with a US $40 gift card to local retail shops to complete the survey. Women who completed the baseline survey (N=288) were randomized to either the intervention arm (n=135) or control arm (n=153). Participants were asked to sign on to Facebook to receive messages through a *private* Facebook group that was invitation only and did not publicly appear as an open group nor available through the search bar menu on Facebook. The intervention and control groups received targeted weekly or biweekly Facebook posts. Electronic engagement was encouraged by a variety of polls, discussions, and raffles.

Unlike commercial Facebook pages, private pages were not eligible for tracking activities (eg, likes, comments) at the time this study was conducted; therefore, methods were limited to manual review of comments, responses, and questions. A manual data collection spreadsheet was initiated and monitored for 2 months but proved too time-consuming to continue with the limited staff supported by the pilot (NIH/NCI R21) grant funding. It was determined to be more important to engage and interact with the participants on Facebook, continuing regular posting of educational messages, and answering questions and comments.

Approximately 4 to 6 weeks postpartum, participants received text invitations and Facebook reminders to complete the postpartum survey. The expected due dates were collected through baseline surveys. Study staff often discovered births, particularly preterm births, through Facebook postings. After delivery and completion of the postpartum assessment, a smaller sample (n=23) of women, family members, and partners were recruited for focus groups or semistructured interviews to qualitatively assess the use of technology as well as the message content.

### Intervention Delivery

The Facebook group breastfeeding messages centered on 5 themes of support: bonding, health and wellness for mother, health and wellness for child, financial impact, and social or lifestyle impact. Eligible participants were randomized into a control Facebook group page that received only these breastfeeding messages or an intervention Facebook group page that received both breastfeeding messaging and breast cancer risk reduction (as it relates to breastfeeding) messaging, including a video presenting this information (Multimedia Appendix 1). All messaging was culturally appropriate for AA women (Multimedia Appendix 2). Participation in the Facebook group pages was intended to engage participants in the topics and viewers were able to *like* posts, comment, ask questions, and contact study staff for more information or if they had any issues or concerns.

As new participants joined the respective Facebook group throughout the study, the 5 topics periodically rotated so that participants engaged in Facebook would be exposed to all 5 topics over approximately 5 weeks. As the topics were presented, the specific content and photos offered new or revised information so that the participants did not see the same content repeatedly.

### Focus Groups and Interviews

In addition to quantitative surveys (for measuring intervention outcomes), focus groups were hosted to discuss participants’ feelings, perceptions, and thoughts on the topic of breastfeeding, messages about breast cancer risk, and participation in the intervention in general. Participants who had completed the study and delivered their babies were invited via text messages and phone calls to attend a focus group. In total, 4 focus groups were hosted at the Roswell Park Comprehensive Cancer Center (2 groups included mothers who participated in the study, and 2 groups included individuals identified as support; eg, family members and partners) by the mothers. These groups were hosted on a Saturday for the convenience of the participants. Although family members and partners did not directly receive Facebook posts, the published research clearly demonstrates that breastfeeding occurs or fails to occur within a larger social context that is directly impacted by the views and experiences of the mothers’ family members and partners. Therefore, the pilot study was designed to explore the social context of the use of Facebook and the messaging with the mothers and their significant others. For example, did the mothers and their supportive family members share the information they learned and how was this received?

All sessions lasted no more than 60 min with the first session starting in the morning and the second session hosted in the afternoon to give participants varying options in which group they would like to participate. The discussion included questions concerning personal perception and relationship with breastfeeding, risk perception of breast cancer, benefits and barriers to breastfeeding, and the use of social media and the messages they received. The groups, including other family members (ie, support), covered the same question content, included an explanation of what the study and intervention addressed, and offered examples of messaging shared with mothers involved in the study. Refreshments were served, and all participants received an incentive of US $30 for participating in the focus group as well as the ability to participate in a free community holiday photography shoot hosted at the cancer center.

Semistructured interviews were used to specifically cover topics that were triggered by the focus group participants in a grounded theory approach and to allow data gathering from male partners and more family members who did not choose to attend focus groups. As some issues related to breastfeeding may be sensitive and mothers and family members may not want to discuss them in a group setting, additional individual interviews were offered with participating mothers and their selected support persons. A total of 12 interviews were conducted, 9 by phone (8 mothers and 1 support participant), and 3 male support member interviews completed in person. The interviews consisted of topics similar to those addressed in the focus groups, such as personal perception and relationship with breastfeeding, risk perception of breast cancer, and benefits and barriers to breastfeeding. Phone interviews averaged 15-20 min and 5-10 min for the in-person interviews. All interviews were conducted by trained study staff and were race concordant. Participants who completed the interviews also received gift card incentives.

All focus groups and interviews were recorded and transcribed. These transcriptions were reviewed and coded by 4 members of the study team. Discrepancies were discussed, and final categorical analyses were determined by consensus. Text coding was sorted into thematic codes and subcodes. Text analysis was validated by discussions with the project staff who were directly interacting with the participants in focus groups, through Facebook and texts.

## Results

### Recruitment

In total, WIC delivered more than 3000 text messages to women enrolled in their mobile messaging platform from which there were 1113 responsive *clicks* on the link to the survey. More than 66.7% (192/288) of the participants were recruited through the WIC e-blasts (Table 2).

Owing to the structure of the delivery of recruitment text through WIC, it was expected to receive fewer *clicks* compared with the text messages delivered, as women could receive the recruitment text from WIC up to 3 times. Through our partnerships with the local health care network, including hospital and prenatal clinics, offices and community-based organizations, Facebook support groups and snowball referrals we received more than 200 incoming text messages initiating the keyword response (from posters, flyers, and personal contacts) to receive the link to begin the survey from which there were 304 *clicks*. It was expected to see more *clicks* compared with incoming text messages from this group, as women engaged and recruited in person would often return to the survey link later to complete the enrollment information, so the conversion to participation rate was higher (78.9% [45/57] compared with 40.7% [192/472] for WIC), although the reach was greater through WIC. Through these combined recruitment efforts, 633 women were assessed for eligibility, with 288 pregnant AA women (103% of the original goal, n=280) successfully enrolled and consented (Table 2). Exclusions included 345 women not meeting inclusion criteria based on race, 29 women declined, 37 women did not provide follow-up contact information for enrollment, 26 women did not complete presurvey assessments, and 9 women gave birth before enrolling in the Facebook group. Of the 288 consented participants, 135 were randomized into the intervention group and 153 were randomized into the control group.

Interestingly, of the women enrolled in the study through local OB/GYN offices and community-based organizations (n=96), 90% (n=86) were currently enrolled in or planning to enroll in WIC services, indicating that the study sample can be expected to be relatively similar from both recruitment sources. Times when women (from all recruitment sources) clicked on the study e-blast invitations, enrolled, and completed surveys on their smartphones were widely distributed: 41.7% (120/288) of surveys were completed between the hours of 7 AM and 3 PM, 54.5% (157/288) of surveys were completed between the hours of 3 PM and 11 PM, and 3.8% (11/288) were completed between 11 PM and 7 AM. Of the 288 participants, 20.5% (n=59) responded to recruitment and enrollment messages on their smartphones *after working hours* between 6 PM and 9 AM.

Overall, 92.4% (266/288) of the mothers stated that they used or knew how to use Facebook, and 99.7% (287/288) used or knew how to text. A total of 96.5% (278/288) of women stated that they were willing to join and use Facebook and text messaging for involvement in the study. Although almost all participants reported knowledge, use, and willingness to engage in Facebook, 39.9% (115/288) of consented participants randomized to the intervention and control arms failed to ever join the assigned private Facebook group. This inadvertently created *true controls* for the study, as 40.0% (54/135) of the breastfeeding plus breast cancer risk (intervention) and 39.2% (60/153) of the breastfeeding-only (control) message groups did not receive any of the educational messages. All participants completed prebirth baseline surveys, and 74.0% (213/288) completed postnatal surveys.

An anecdotal finding as women had their babies was that 60 new mothers from both Facebook groups became more active and engaged with the topics of breastfeeding and newborn care and requested to be able to continue to communicate on their Facebook page (study participants were considered *complete* and removed from the study protocol and follow-up once they completed their postnatal survey about 4- 8 weeks after the birth of their infants). In response to this, the study staff set up a third Facebook group for women after they had completed the postnatal survey and study protocol, but it was just an open group without designed study messages. As this was not part of the original study design and budget, no further data collection or postnatal messaging was planned.

**Table 2:** Recruitment sources for participants.

Sources	Recruited, n (%)	Enrolled and randomized, n (%)	Conversion rate, %
WIC^a^ e-blast	472 (74.6)	192 (66.7)	40.7
Obstetrics and gynecology office	57 (9.0)	45 (15.6)	78.9
Hospital recruitment	13 (2.1)	6 (2.1)	46.2
Facebook recruitment	44 (7.0)	16 (5.6)	36.4
WIC office recruitment	14 (2.2)	8 (2.8)	57.1
Community-based organization	24 (3.8)	14 (4.9)	58.3
Friend referral	9 (1.4)	7 (2.4)	77.8
Total	633 (100.0)	288 (100.0)	45.5

^a^WIC: Women, Infants, and Children.

### Focus Group and Interview Participants

Of the 10 female participants who attended the focus groups, 6 participant mothers and 4 family or friend support individuals (2 mothers and 2 friends), all mothers reported breastfeeding for at least three weeks. The participants’ average age was 33.3 years (median 29.0, SD 10.4), 4 participants reported working full time or part time, 3 participants completed high school or had a GED certificate, 4 participants completed some or all college, 7 participants reported earning US $20,000 or less in household income, and all participants reported being insured. Of the 9 female participants who completed interviews over the phone, the mean age of mothers was 28.4 years, 6 had never married, 9 reported completing high school or more, 3 reported working full time, and 6 earned less than US $20,000. We did not collect demographics for the 3 male partners.

Of the female participants in focus groups and interviews, 9 participants were aged >25 years and were often more experienced with pregnancy and breastfeeding than the total study sample. Participants with prior breastfeeding experience were very positive about breastfeeding, social media messages, and supportive of any or all efforts to increase breastfeeding. Other less-experienced participants reported knowing very little before the intervention, such as, “Didn’t know much before…,” “Learned about how it helped the baby,” and “Was just going to see how it went.” Another inexperienced mother demonstrated the limitations of education alone, stating, “What I saw made it look easy, and that wasn’t true—not for me.” The participants did report a litany of the benefits they learned about breastfeeding (eg, brain development, saving money, benefits to the mother, bonding, and lowering the risk of breast cancer). It was notable that several mothers reported memorable messages about breastfeeding rights and issues of “to cover-up or not cover-up when breastfeeding in public.”

In response to the use of Facebook, almost all focus groups and interview participants would have liked to stay in the group longer, possibly share posts, or to have had an ongoing Facebook support group postpartum. There was a general sentiment that “I would have liked to invite friends into the group;” “I wanted to share posts with friends in other groups;” be allowed “to share content with other groups and friends;” “I would have loved to invite others!” The participants also would like to have had access to more videos on the Facebook pages. All the women expressed support for the title and logo of the Facebook group, “Black Breasts Matter.” Inquiries about the logo on Facebook also received positive responses. “It was wonderful because you’re supporting Black women;” “Rings a bell;” “Loved it!” Several participants stated that they do not see very much media or information specific to Black women and breastfeeding: “I think that Caucasian people actually do it [breastfeed] more than black women, so that gets us motivated to do something that we’re not so familiar doing” and “I liked it because it had to do with blacks…you know it was more for like black mothers and it made me feel comfortable.” (Notably, the tag to initially enroll in the study was “Our Breasts Matter” in deference to the New York State Health Department concerns showing any racial exclusion with the study. It was not until a participant met qualifications and was consented that they saw the “Black Breasts Matter” logo. Ineligible White participants were routed to other breastfeeding resources.) Interestingly, one participant shared the perspective that Black women’s breast milk is superior to other mothers’ milk and believed this could be empowering other Black women to breastfeed. Breast cancer risk information and messaging about potential risk reduction from breastfeeding for those in the intervention group was commented on as being *impactful* and new information for most of the women, and they wanted to share this information with family and others.

## Discussion

### Principal Findings

This study describes the use of social media and electronic platforms to effectively recruit and engage pregnant AA women into an intervention study focused on educating about the benefits of breastfeeding and the potential to reduce breast cancer risks. The specifics of how smartphone and social media apps can create a social and educational space as well as a novel approach for discussing breastfeeding as a form of breast cancer risk reduction, specifically for AA women, are described. The process evaluation illustrates various strengths and limitations of methods and recruitment processes for this demographic of participants and suggests ideas for future studies incorporating smartphone and social media use.

The overall strengths of the study and the methods employed included the successful (18 months) recruitment of 288 pregnant AA women into the study and the effectiveness of electronic consent forms and surveys. More than 58.3% (168/288) of participants explored study participation and eligibility and consented and completed surveys during evening hours (after 3 PM). Almost 20.5% (59/288) of participants completed study tasks during nontraditional working hours for study staff (ie, 6 PM-9 AM). The ability of participants to complete surveys and read messages by smartphone at any time of the day or night, depending on their schedules and needs, was an advantage for the study staff and participants and was less challenging than recruiting for and scheduling the in-person focus groups or interviews for this study. This recruitment process was also more effective than attempts in a previous study in Buffalo in collaboration with Planned Parenthood that had very poor results collecting in-person survey data during daytime working hours from this demographic of younger women regarding tobacco use and cervical cancer risk [20].

Of the recruitment sources explored, WIC e-blast had the greatest reach and proportion of participants enrolled (192/288, 66.7%) as well as being able to contact potential participants multiple times in a cost-effective way. In-person recruitment using keyword responses at prenatal clinics and community-based organizations resulted in a lower proportion of enrollments (approximately 30%), but the conversion rate for these in-person and especially the snowball friend referrals (7/9, 77.8%) was high. These more direct methods were also more time-consuming and labor-intensive (Table 2).

It was notable that the participants strongly endorsed and welcomed the racial and cultural tailoring of the messages, photos, and issues presented in the Facebook intervention. Qualitative responses included pride and opportunities for the promotion of breastfeeding for enhancing Black infants’ and mothers’ health and appreciation for focusing on their needs and issues at the policy level. As the lowest rates of breastfeeding are among AA women and many of the barriers, including racially biased health care [1] and negative social contextual issues disproportionately impact AA women, it could be important to address these issues in future intervention approaches. Moreover, incorporating racially specific messages may enhance self-efficacy by AA mothers to address policies and feel greater support through social media.

### Future Studies and Limitations

An important consideration specifically for studies incorporating Facebook is that there are operational differences for a private Facebook group versus more commercial, *business*, or even personal Facebook accounts. A private Facebook page operates in a much more limited fashion than an organic, individually based, or business-based Facebook page that includes individuals familiar with one another or with some type of shared experiences. The fact that all participants were pregnant helped to focus and engage some women, but they were not naturally familiar with one another, and the general trust and sharing activities in the study Facebook pages were more limited. As time progressed, women seemed to become more familiar with one another, especially as they gave birth and were faced with issues that they wanted to share, and this reinforces that support and evaluation of social media activity postnatally should be included in future research. The fact that 60 mothers were interested in having more education and interaction after their babies were born and they had completed the study demonstrates that addressing the issues and challenges of new mothers, including those of breastfeeding, often gain much greater awareness with the immediacy of needs. This further reinforces the theory that *teachable moments* and *need to know* timing is important for adult learners. The comments by participants also indicate limitations in the nature of the private Facebook groups, as the information on their group pages was limited to sharing only with study participants. Participants could not electronically *share* with other family and friends or Facebook pages. This may have limited activity, ability to influence their own social network, as well as the responses and conversations about the postings.

One limitation of the study recruitment process in collaboration with New York State WIC was the fact that the New York State Department of Health limited all research to be done with individuals aged ≥18 years. This was not known by the study team before the initiation of the study. As up to 2% of births among AA women are in women aged <18 years and the younger the age at first birth, the more births they are likely to have, resulting in potentially higher risks for TNBC, it was important to include younger women [21]. The study IRB approval process allowed recruitment of women aged ≥14 years; however, it was challenging to reach and recruit these younger women without the WIC recruitment process, even within the local OB/GYN practices.

National corporate changes in technology security issues with Facebook are issues that may impact participation in a study such as this one. With the use of social media comes the constant alteration, upgrades, and changes to the operational use of the app as well as features that are accessible through the app that may leave users vulnerable. In 2018, as the study was winding down, Facebook publicly faced two major scandals concerning the use of private information of its users. It was publicized that Facebook faced a breach and attack on its computer network, which in turn left the personal information of nearly 50 million users available as well as access to their accounts. Although discussions in community settings occurred about the safety of Facebook, we did not see a decline in recruitment after the announcement of the breach, but we did have additional challenges.

Challenges and limitations of the social media methodology included the fact that about one-third (n=114) of the participants never chose to sign on to and receive any messaging from the private Facebook groups, basically creating a *true* control with no messaging about breastfeeding. It was unclear why participants failed to sign on, as survey data demonstrated a high proportion (>92%) of participants had knowledge of and used Facebook personally. This may have been the result of a lack of trust or unfamiliarity with the use and practices of a private Facebook account. This reinforces the challenges of using Facebook for this clientele, as it may be waning in popularity as a communication platform with this demographic.

At the time of the study, Facebook did not have a feature to be able to collect analytical metrics from Facebook groups but rather only activated this feature for business pages. One of the more recent advancements made is “Group Insights,” which now allows the generation of reports from a created group including metrics such as the growth of total members, pending members, approved requests, engagement through posts, comments, reactions, activity status, and member contribution ranking members who contribute more than average members. Unfortunately, this advancement could not be applied to an already established group but is a current feature available for future intervention research.

Monitoring the use of Facebook was another limitation that we encountered. The culture of social media is based on immediate responses, new content that is updated frequently, and 24-hour access. The staff implementing this study was small (2 part-time staff); therefore, it was challenging to meet the demands for posting new messaging and responding to study participants’ comments and questions quickly compared with other social media groups who have dedicated staff members posting and monitoring content. Feedback from study participants (eg, posting more videos) indicated that they would have liked more production and were more engaged within the Facebook group pages by having more access to study staff through Facebook Live and web-based question-and-answer sessions. In addition, when using a social media platform such as Facebook for research study purposes, there is a learning curve for what information can be accessed and tracked and best practices on how to do so. The study staff learned that there are restrictions put in place by Facebook that did not allow them to automatically track and extract a study participant’s frequency of participation within the group. Some of these limitations are different for private accounts versus commercial or personal accounts.

### Conclusions

Educating pregnant AA women about the positive attributes of breastfeeding and potential benefits for breast cancer risk reduction is countered by strong circumstantial and time constraints for many who may still be in school and are still more engaged in their age cohort activities and interests. The options for providing messaging and resources through social media to reinforce breastfeeding and possibly provide lactation support hold promise. Convenience is a major attribute of social media for this demographic and worthy of further research as an educational tool.

The use of text messaging platforms such as Telerivet in combination with survey collection software holds promise as an effective tool for electronic recruitment for populations in which convenience is a big factor of participants into a study and allows for larger volumes of people to be reached instantly as well as data collection. Social networking platforms can be an effective tool for the dissemination and engagement of education information cohorts but require a group of moderators dedicated to the management of the tool, including timely response to comments and questions, consistency of posting as well as in-person support for a behavior such as lactation counselors for breastfeeding.

Methodologically, this social media approach holds promise for both recruitment and conduct of future breastfeeding intervention studies. Repeat studies may consider extending Facebook access pre- and postnatally, new metric analysis such as “Group Insight,” and to consider links to additional, live lactation support services, including racially concordant opportunities. Further analysis and outcomes may be a call to current providers of peer support such as WIC to implement a web-based system to support the mission of WIC and peer counselors.

## Appendix

Multimedia Appendix 1 Youtube video link for “African American women reduce risk of breast cancer by breastfeeding”.

Multimedia Appendix 2 Messaging by topic.

## Abbreviations

GED: general educational development
IRB: institutional review board
NCI: National Cancer Institute
NIH: National Institutes of Health
OB/GYN: obstetrics and gynecology
TNBC: triple-negative breast cancer
WIC: Women, Infants, and Children
