# Supplementary figures and images for: #BlackBreastsMatter: Process Evaluation of Recruitment and Engagement of Pregnant African American Women for a Social Media Intervention Study to Increase Breastfeeding
Source: J Med Internet Res. 2020 Aug 10;22(8):e16239. doi: 10.2196/16239 (PMC7445612; doi:10.2196/16239)

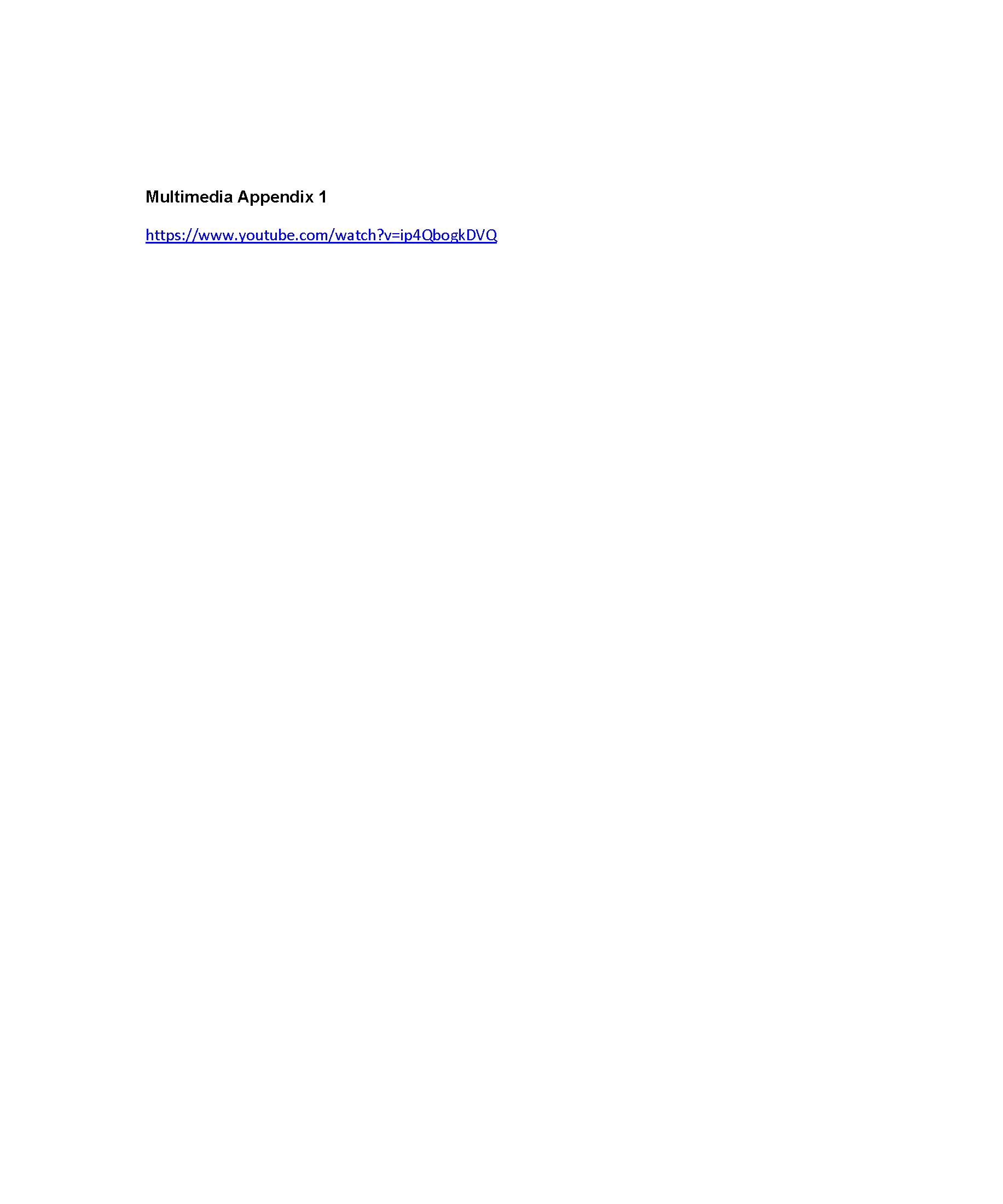

Supplement: Multimedia Appendix 1 [file jmir_v22i8e16239_app1.png]
