# Supplementary material for: #BlackBreastsMatter: Process Evaluation of Recruitment and Engagement of Pregnant African American Women for a Social Media Intervention Study to Increase Breastfeeding
Source: J Med Internet Res. 2020 Aug 10;22(8):e16239. doi: 10.2196/16239 (PMC7445612; doi:10.2196/16239)
Supplement: Multimedia Appendix 2 [file jmir_v22i8e16239_app2.pdf]

Table 2. Messaging by Topic

Bonding	Nikia Clark shared a photo.<br>April 16, 2018 · Add Topics<br>After delivery, it's important to breastfeed and bond with your baby skin-to-skin as soon as possible.<br>#blackbreastsmatter<br>#breastfeedingmatters<br>#bondingwithyourbabymatters<br>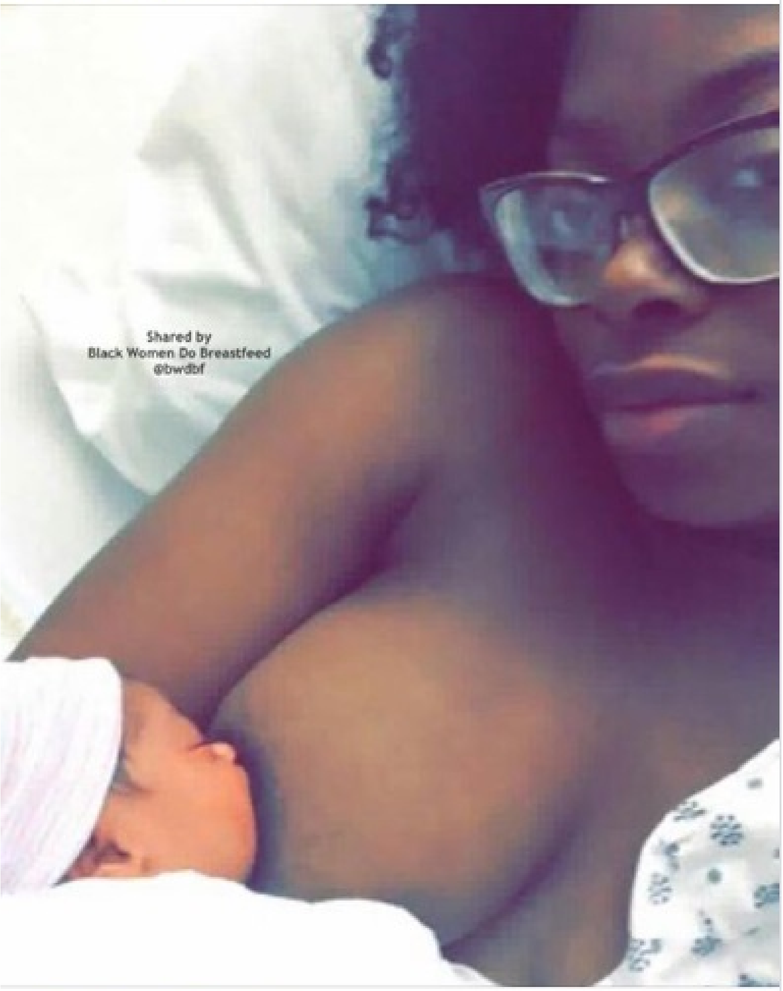<br><br>Black Women Do Breastfeed<br>March 6, 2018<br>"Not even 24 hours old and she's already a pro! I'm Looking forward to a nice long journey of bonding with this angel!"<br>#bwdbf #wedothis	

Nutrition<br><br>(Baby & Mom	Nikia Clark<br>April 3, 2018 · Add Topics<br>Eating a well balanced diet and drinking plenty of water will help boost your energy and increase your milk supply when you breastfeed! What are some of your favorite healthy snacks and foods? Comment below or add a picture.<br>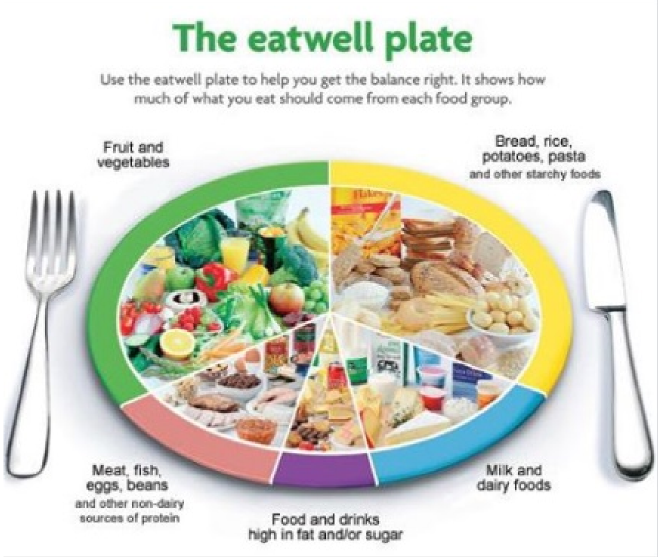<br><br>http://www.fao.org/3/a-as838e.pdf
Health &<br><br>Wellness<br><br>(Baby &<br><br>Mom)	Nikia Clark<br>December 1, 2018 · Add Topics<br>Who feels like they know all the benefits breastfeeding has for the mother and baby?<br>One benefit for the mom is BREASTFEEDING REDUCES STRESS. Can you name more benefits?<br>#blackbreastsmatter<br>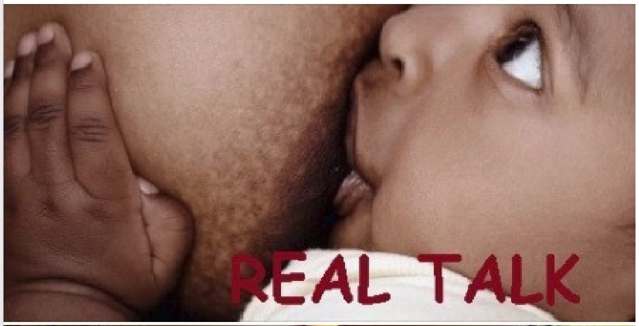<br>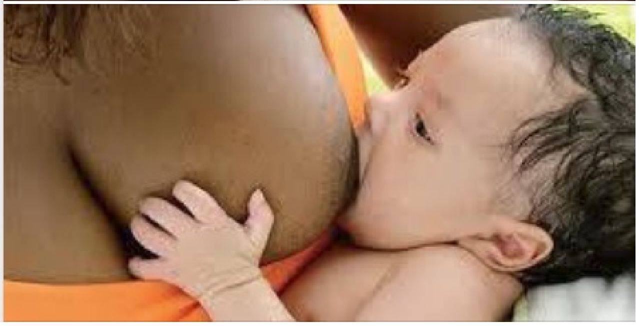<br><br>http://mochamanual.com/bb/tips/byb/

Finances /<br><br>Money<br><br>Saving	Nikia Clark shared a link.<br>June 11, 2018 · Add Topics<br>Listen to this video and hear what the experts say about breastfeeding. The top 2 reasons to breastfeed are: 1. Healthiest choice for the baby and<br>2. It saves money<br>What is the most important reason for you?<br>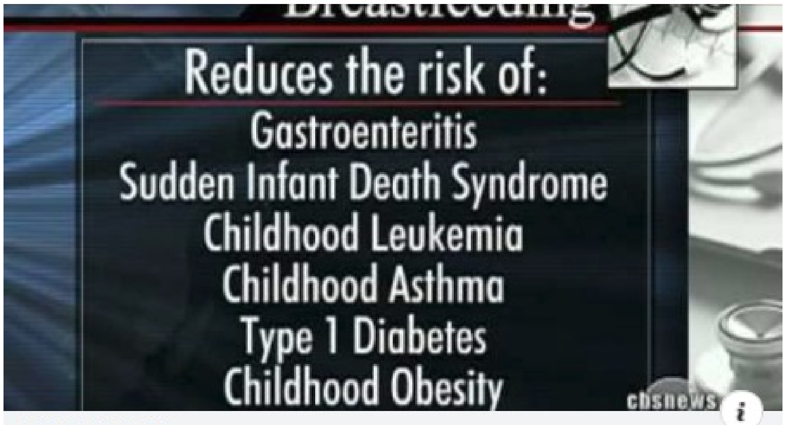<br>YOUTUBE.COM<br>**Breast Feeding Saves Lives, Money**<br>A new report shows that breast feeding can save nearly 900 infants and $...<br><br>https://www.youtube.com/watch?v=jLKPi-M76VY
Support for<br><br>the mom	Nikia Clark shared a link.<br>March 12, 2018 · Add Topics<br>The #1 reason women stop breastfeeding in the first 3 weeks after giving birth is the lack of support.<br>This week we will spend some time talking about challenges, resources and advice about getting the support you need to breastfeed successfully. ALL YOUR COMMENTS ARE WELCOME!<br>This video talks about dads but the info is useful for whoever is your support person. Let me know what you think!<br>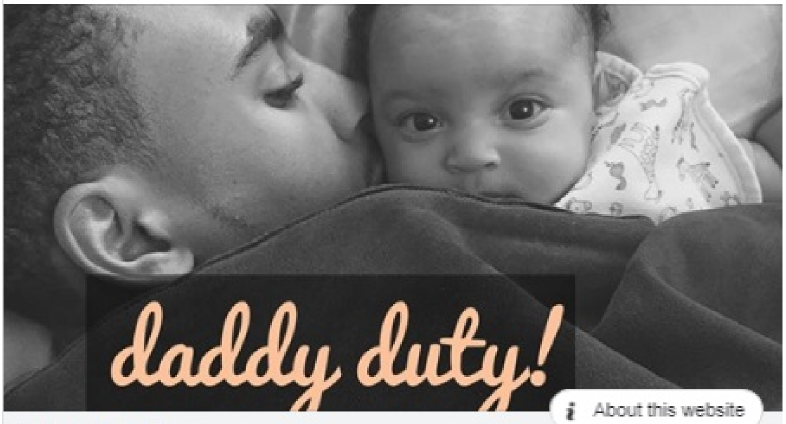<br>About this website<br>YOUTUBE.COM<br>**How Dads Can Help Breastfeeding Mothers**<br>Welcome baaaack! Today we talking all about helping out breastfeeding...

History of Breastfeeding in the African American community	Nikia Clark shared a link.<br>June 11, 2018 · Add Topics<br>Did you ever think of why black mothers are less able to breastfeed? What are some challenges you might have that will make it hard to breastfeed?<br>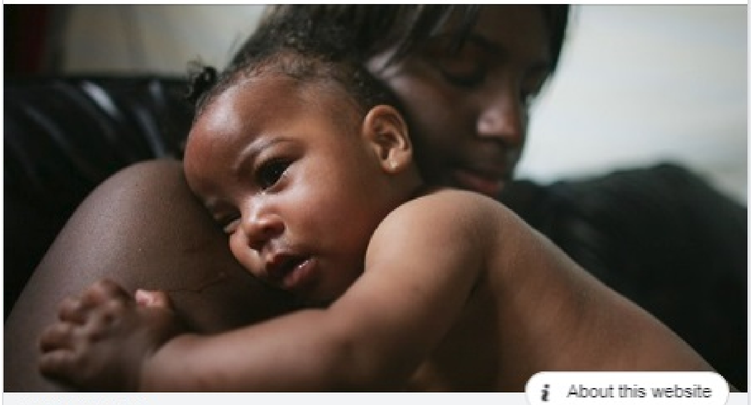<br>About this website<br>ROMPER.COM<br>**The Unjust Reason Black Mothers Are Less Able To Breastfeed**
Challenges and Concerns	Nikia Clark shared a link.<br>October 2, 2018 · Add Topics<br>The proper latch is important!<br>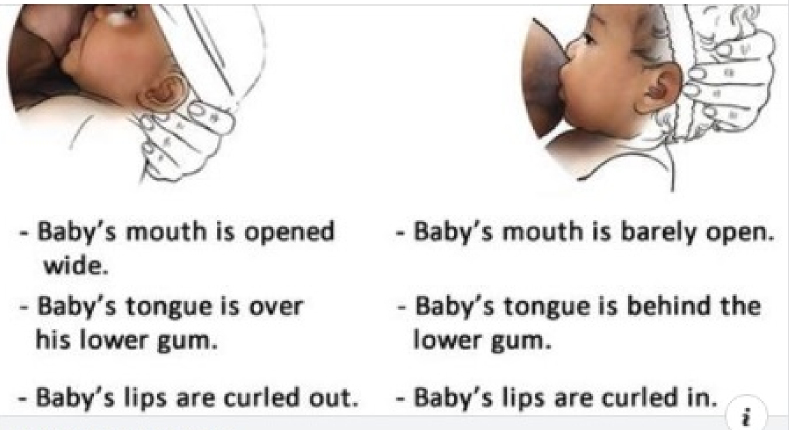<br><br>IMAGES.GOOGLE.COM<br>**Image: Similiar Newborn Latch Breastfeeding Babies Keywords**
